# Unraveling the unknown areas of the human metabolome: the role of infrared ion spectroscopy

**DOI:** 10.1007/s10545-018-0161-8

**Published:** 2018-03-19

**Authors:** Jonathan Martens, Giel Berden, Herman Bentlage, Karlien L. M. Coene, Udo F. Engelke, David Wishart, Monique van Scherpenzeel, Leo A. J. Kluijtmans, Ron A. Wevers, Jos Oomens

**Affiliations:** 1 Radboud University, Institute for Molecules and Materials, FELIX Laboratory, Toernooiveld 7c, 6525ED Nijmegen, The Netherlands; 2 Department of Laboratory Medicine, Translational Metabolic Laboratory, Radboud University Medical Center, Geert Groote Plein Zuid 10, 6525 GA Nijmegen, The Netherlands; 3 Departments of Computing Science and Biological Sciences, University of Alberta, Edmonton, AB Canada; 4 Van’t Hoff Institute for Molecular Sciences, University of Amsterdam, Science Park 904, 1098 XH Amsterdam, The Netherlands

**Keywords:** Infrared ion spectroscopy, Feature identification, Biomarkers, Molecular structure analysis, Inborn errors of metabolism, HMDB, Computational chemistry

## Abstract

The identification of molecular biomarkers is critical for diagnosing and treating patients and for establishing a fundamental understanding of the pathophysiology and underlying biochemistry of inborn errors of metabolism. Currently, liquid chromatography/high-resolution mass spectrometry and nuclear magnetic resonance spectroscopy are the principle methods used for biomarker research and for structural elucidation of small molecules in patient body fluids. While both are powerful techniques, several limitations exist that often make the identification of unknown compounds challenging. Here, we describe how infrared ion spectroscopy has the potential to be a valuable orthogonal technique that provides highly-specific molecular structure information while maintaining ultra-high sensitivity. Here, we characterize and distinguish two well-known biomarkers of inborn errors of metabolism, glutaric acid for glutaric aciduria and ethylmalonic acid for short-chain acyl-CoA dehydrogenase deficiency, using infrared ion spectroscopy. In contrast to tandem mass spectra, in which ion fragments can hardly be predicted, we show that the prediction of an IR spectrum allows reference-free identification in the case that standard compounds are either commercially or synthetically unavailable. Finally, we illustrate how functional group information can be obtained from an IR spectrum for an unknown and how this is valuable information to, for example, narrow down a list of candidate structures resulting from a database query. Early diagnosis in inborn errors of metabolism is crucial for enabling treatment and depends on the identification of biomarkers specific for the disorder. Infrared ion spectroscopy has the potential to play a pivotal role in the identification of challenging biomarkers.

## Introduction

Many biomarkers of inherited metabolic diseases can be diagnosed in high-throughput targeted analyses that are often sufficiently routine for large scale screening. However, the development of such protocols requires the knowledge of diagnostic biomarkers which, by definition, must be first identified in (un)targeted analyses of patient body fluids. Currently, (ultra) high performance liquid chromatography/high-resolution (tandem) mass spectrometry ((U)HPLC/MS^(n)^) is one of the most successful techniques for untargeted profiling of patient body fluids in order to identify small molecule metabolites that correlate with, and are specific to, a given disorder and can thus be used as biomarkers. Another untargeted approach is nuclear magnetic resonance (NMR) spectroscopy, though this technique is limited to metabolites that are above the low micromolar (μM) range and is thus blind to a significant portion of physiologically-relevant concentrations. LC/MS^(n)^, on the other hand, is an ultrasensitive technique and can routinely detect thousands of compounds in a body fluid down to the low nanomolar (nM) range, though it lacks the specificity to molecular structure that is often necessary to distinguish isobaric compounds. Thus, the techniques are complementary in the sense that LC/MS^(n)^ is very good at detecting mass/charge (m/z) features in a sample that correlate with a particular disorder and NMR is very good at identifying the molecular structures of unknowns. This is illustrated in the recent identification of a new inborn error of metabolism (IEM), NANS deficiency (van Karnebeek et al 2016). The metabolite identified as a biomarker of the disease was detected by untargeted metabolite screening using LC/QTOF-MS, where its concentration was well within the sensitivity of the technique, but could not be unambiguously identified from its structural analogues on the basis of retention time, m/z ratio or fragmentation behavior. Thus, NMR was used to identify the molecular structure to correspond to N-acetylmannosamine by comparison to reference material; however, it was close to the limits of sensitivity for NMR. Thus, in order to push the limits of biomarker discovery, advances in analytical methods that combine both extreme sensitivity and highly specific molecular structure information have a critical role. Here, we outline the potential that infrared ion spectroscopy (IR-IS) has to fill such a role in the state-of-the-art toolbox of metabolomics researchers.

Infrared (vibrational) spectroscopy is a well-established technique that is often used in the mid-IR region, or the vibrational fingerprint region, to probe the fundamental vibrations of molecules. This technique is often used, for example, in the organic chemistry laboratory, to determine the presence or absence of particular functional groups in a molecule in either the solid, liquid or gas phase. This is possible because the IR features of different functional groups are found at characteristic (and very often unique) IR frequencies and make it possible to generate a fingerprint or signature of a particular molecule if a range of IR frequencies (for example, 1800–600 cm^−1^) is sampled. These types of measurements are based on detecting the extent of attenuation of the IR light after it passes through a sample where photons are absorbed when they are at resonant frequencies with one of the vibrations of the molecule. Infrared ion spectroscopy (IR-IS) is the combination of ion trap mass spectrometry and infrared spectroscopy, selectively generating an IR spectrum for any mass-isolated ion in the MS spectrum (see figures below). In contrast to the standard IR absorption spectroscopy described above, IR-IS detects the absorption of IR photons on the basis of the photofragmentation they induce in a set of precursor ions trapped in a mass spectrometer. This approach exploits the mass spectrometer as an extremely sensitive detector and is necessary because the number densities of ions (of the same charge) required to produce a measurable attenuation of the IR light cannot be reached. Measurements are conducted directly inside the mass spectrometer and thus maintain full sensitivity and require no additional experiments outside of the mass spectrometer. The IR spectrum of a given m/z feature, however, adds an orthogonal level of molecular structure information in the form of an IR fingerprint. IR-IS has evolved in little more than a decade from an experimental technique in academic labs studying fundamental molecular physics (Lemaire et al 2002; Aleese et al 2006; Oomens et al 2006; Fridgen 2009; Polfer and Oomens 2009) to its current state, in which it is a demonstrated (bio)analytical technique for the identification of small molecules in complex mixtures (Martens et al 2017a, b), though still limited to user facilities or state-of-the-art laboratories housing the most advanced tuneable infrared laser technology. In the past decade, IR-IS has been used to identify and characterize the molecular structures of many classes of chemical compounds including amino acids (Polfer et al 2005, 2006a; Correia et al 2008; Rodgers et al 2008; Oomens and Steill 2009; Scuderi et al 2011), nucleotides and bases (Salpin et al 2007; Chiavarino et al 2013), peptides and proteins (Balaj et al 2008; Polfer et al 2008; Yoon et al 2008; Fukui and Takahashi 2012; Martens et al 2012, 2015, 2016a; Stedwell et al 2012; Scuderi et al 2015; Dunbar et al 2017), saccharides (Martens et al 2017b; Polfer et al 2006b; Cagmat et al 2010; Contreras et al 2012; Schindler et al 2014, 2017), neurotransmitters (Lagutschenkov et al 2010, 2011), and a variety of other mainly small organic compounds and reaction products (Martens et al 2017a; MacAleese and Maître 2007; Rummel et al 2011; De Petris et al 2013, 2016b; Warnke et al 2015; Cismesia et al 2016; Seo et al 2016; Schäfer et al 2017; Gorlova et al 2017). Here we outline various ways IR-IS can currently be utilized by the metabolomics community. As an example, we focus largely on a biomarker of the IEM glutaric aciduria (GA), glutaric acid, and an isobaric metabolite, ethylmalonic acid (itself a biomarker for, among others, the IEM short-chain acyl-CoA dehydrogenase deficiency, (SCAD)). As well, we demonstrate an aspect of IR-IS that sets it apart from other tandem MS techniques; IR spectra can accurately and routinely be calculated in silico for entirely arbitrary molecular structures, unlocking the potential for reference standard free identification. As an additional example, we use a set of saccharides to illustrate how IR-IS can be applied to identifying enantiomeric compounds. Finally, in the same set of compounds we show how functional group information can be obtained for a complete unknown, which is valuable information for, as an example, narrowing down a list of candidate structures resulting from a database search result. This is especially relevant for the application of untargeted metabolomics in body fluid samples in individual patients. In such analyses many features occur that cannot be assigned by current database searches thus hampering further progression of such approaches in current clinical practice.

IR-IS is a potentially valuable new approach to the challenge of identifying the many unknowns that arise from untargeted metabolomics analyses of body fluids. Enabling annotation of the most important “unknown features” in such body fluid analyses may reveal previously unrecognized IEMs and is critical for the field to make steps forward in using untargeted metabolomics in research and diagnostics of IEMs. This article shows that, in the view of the authors, IR-IS is, already in its current state, a valuable and accessible tool for molecular identification to the metabolomics community and it outlines several paths forward to expand this role. We discuss accessibility of the IR-IS technique both in terms of sample submission to laboratories operating as international user facilities, and possibilities arising from recent technological advances enabling table-top IR-IS installations in state-of-the-art (bio)analytical laboratories.

## Methods

### Infrared ion spectroscopy

All IR-IS measurements were performed in a modified quadrupole ion trap mass spectrometer (Bruker, amaZon Speed ETD) coupled to the IR beam line of the FELIX free electron laser. The FELIX laboratory (http://www.ru.nl/felix/) is an international user facility and beam time is awarded through calls for proposals. Details of hardware and software modifications to the mass spectrometer have been reported previously (Martens et al 2016c). Anonymized body fluid samples were diluted to give analyte concentrations of ~10^−7^ M in order to sufficiently dilute the higher concentration components of the sample. The diluted samples were directly electrosprayed (+ESI, -ESI). Reference IR measurements were generated from solutions of 10^−7^–10^−8^ M (in 50:50 acetonitrile: H_2_O) of the appropriate reference standard. Reference standards and electrospray solvents were obtained from Sigma-Aldrich.

For these experiments, the FELIX free electron laser was operated in the 600–1850 cm^−1^ range, giving 5 μs macropulses (at a 10 Hz repetition rate) of 30–60 mJ per pulse (bandwidth is ~0.4% of the center frequency). When the frequency of the IR laser is resonant with a vibration in the population of trapped ions absorption occurs and leads to an increase in the internal energy of the ion. Facilitated by intramolecular vibrational redistribution of the absorbed energy, the ions dissociate once they reach the limit of the dissociation channel with the lowest barrier. Typically, after a single macropulse, dissociation occurs providing a frequency-dependent fragment ion m/z in the mass spectrum. By relating the parent and fragment ion intensities (yield = ΣI (fragment ions)/ΣI (all ions)) to the IR frequency, an infrared spectrum is generated. The yield at each IR point is obtained from four to eight averaged mass spectra and is linearly corrected for laser power. Note that the FELIX pulse energy can be decreased with calibrated attenuators in order to prevent complete depletion of the parent ion population. The frequency is calibrated online using a grating spectrometer.

### Quantum chemical calculations

Molecular structures manually defined (based on chemical intuition and known gas-phase ion chemistry) were optimized for each ion of interest at the MP2/aug-cc-pVDZ level in the commercially available software package Gaussian09 (Frisch et al 2009). For each optimized molecular geometry, anharmonic vibrational frequencies were computed and are presented unscaled. Infrared absorption lines were broadened using a Gaussian line shape of (FWHM 25 cm^−1^) to facilitate comparison with the experimental spectra.

## Results

**Fig. 1:**
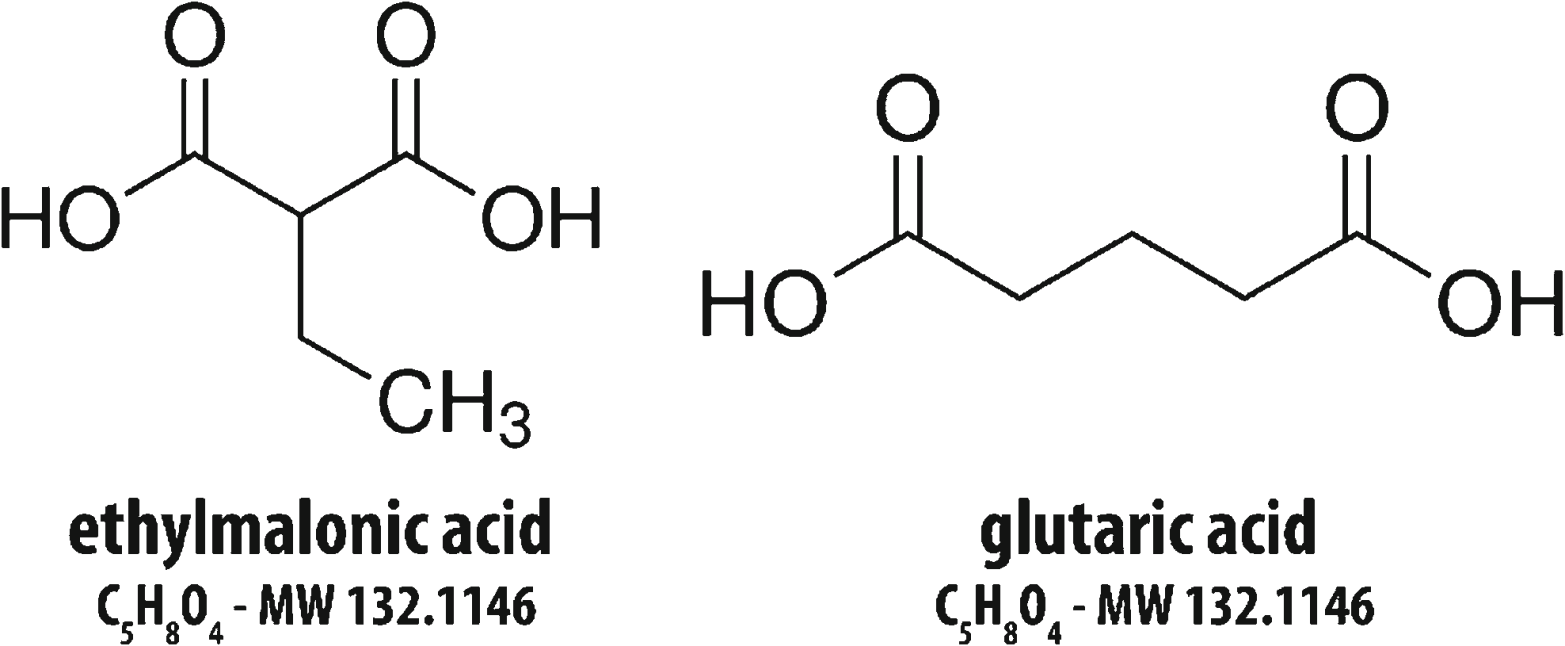
**Structures of isobaric metabolites ethylmalonic acid and glutaric acid.** These compounds are dicarboxylic acids differing by the branched or linear group connecting the two acid moieties

Glutaric acid and ethylmalonic acid are two well-known small organic acid metabolites (depicted in Fig. 1) that can be ionized in both positive and negative mode electrospray ionization (+ESI, -ESI) by protonation or deprotonation, respectively.

**Fig. 2:**
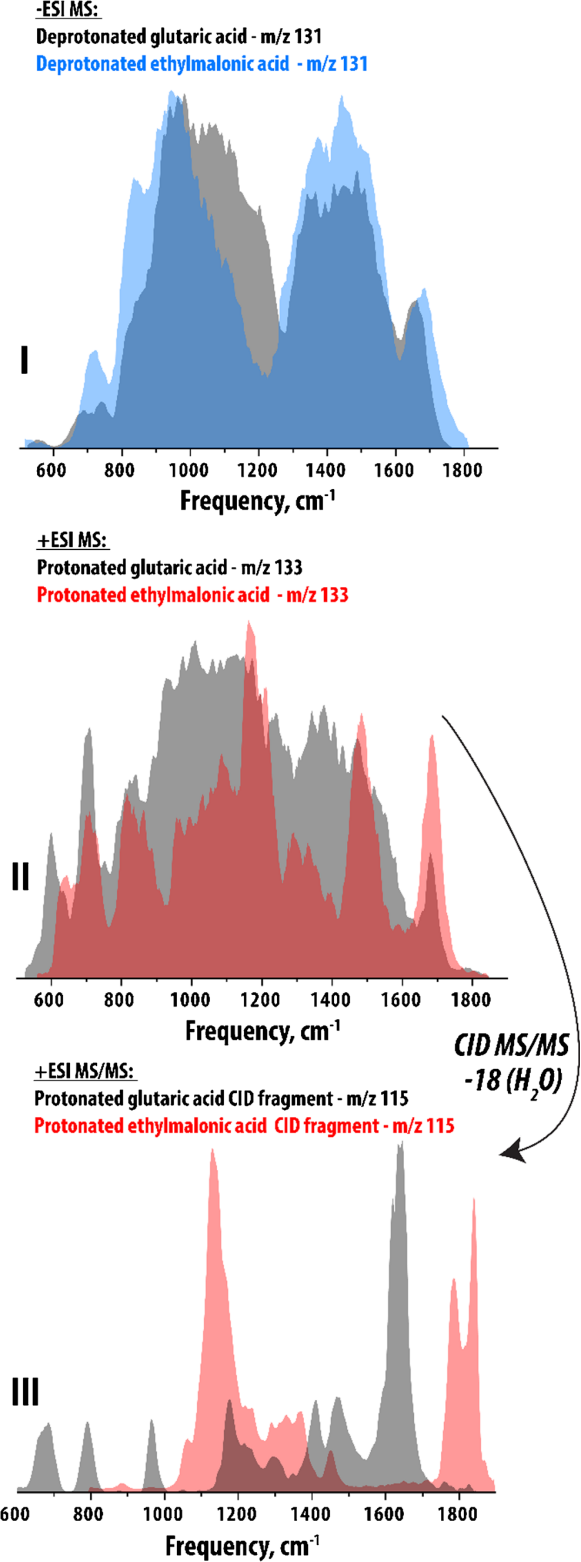
**Various IR spectra of glutaric acid and ethylmalonic acid.** Panel I presents the IR spectra of deprotonated glutaric acid (m/z 131 black) and ethylmalonic acid (m/z 131, blue). Panel II presents the IR spectra of protonated glutaric acid (m/z 133, black) and ethylmalonic acid (m/z 133, red). Panel III presents the IR spectra of the re-isolated CID fragments (in both cases m/z 115, neutral loss of mass 18 - H_2_O) from protonated glutaric acid (black) and ethylmalonic acid (red)

Distinguishing these two compounds using standard reversed-phase HPLC/(tandem)MS is a challenge as both their retention times and their MS/MS fragmentation behavior are very similar. Using reference standards for these two compounds, we have generated IR spectra of both their deprotonated and protonated ions in order to explore potential differences arising from the different structures and gas-phase conformations, illustrated in panels I and II of Fig. 2, respectively. In both cases, the ions of the two compounds are clearly distinguishable by IR-IS, however, we observe relatively broad bands that are not very clearly resolved in terms of frequency, especially for the deprotonated ions (panel I).

The gas-phase conformations of these protonated/deprotonated diacids likely involves a hydrogen bond between the ionized acid group and the neutral group. The difference in length of the carbon backbone connecting the two acid groups will influence the nature of the interaction between the two groups, especially upon ionization, in which case the interaction becomes stronger. This effect has a clear influence on the IR spectra, illustrated in Fig. 2 (panels I and II), though in both cases all spectra exhibit quite broad peaks that are not well resolved from each other. In order to gain a second-level identification of these two compounds we can do MS/MS fragmentation of the precursor ions (shown here for only the protonated ions, m/z 133 in both cases) and re-isolate one of the dominant fragments, in this case loss of neutral H_2_O (mass 18). Subsequently, we measured the IR spectra of the isolated fragment ions (m/z 115 in both cases). These results are presented in panel III, showing again clear distinction between the glutaric acid-derived fragment ion (black) and its ethylmalonic acid derived analogue. In this case, however, we observe clearly different IR spectra for the two ions, both exhibiting sharp and well resolved bands over the entire vibrational fingerprint region (600–1900 cm^−1^), with multiple frequencies that are resonant with vibrations in only one species and thus independently diagnostic.

**Fig. 3:**
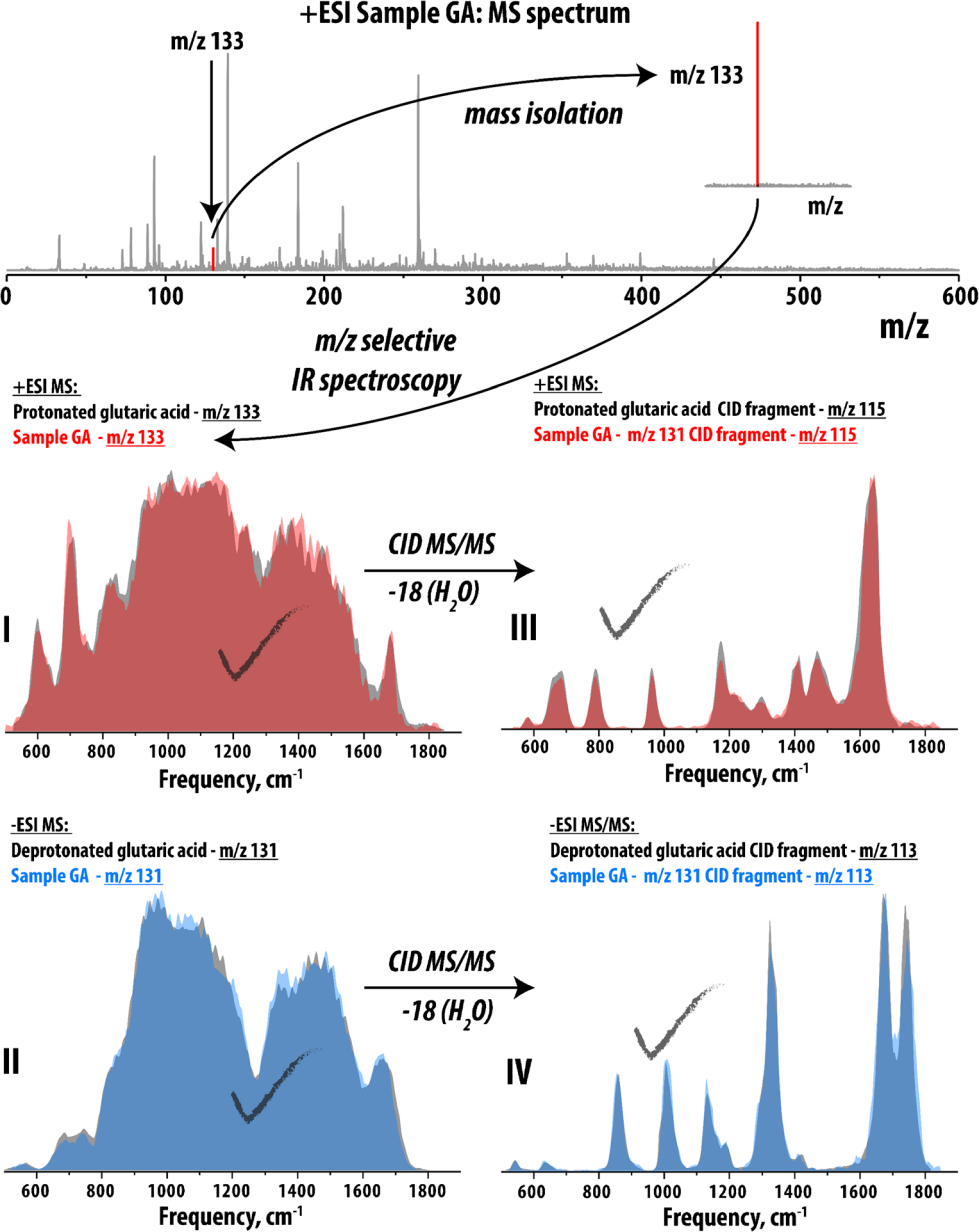
**IR spectra of four glutaric acid derived ions and IR spectra of the corresponding m/z features from a urine sample of a patient with GA.** Above the figure, the full MS spectrum in +ESI mode of the GA sample. An ion at m/z 133 is identified, mass isolated, and trapped in the MS. In this manner, IR-IS generates mass-selective IR spectra for any ion observed in an MS or MS/MS (not schematically illustrated here) spectrum. Panel I presents the IR spectra of protonated glutaric acid (m/z 133 black) and m/z 133 from a GA patient sample (red). Panel II presents the IR spectra of deprotonated glutaric acid (m/z 131, black) and m/z 131 from a GA patient sample (red). Panel III presents the IR spectra of the re-isolated CID fragment (m/z 115, neutral loss of mass 18) from protonated glutaric acid (black) and m/z 115, neutral loss of mass 18 from m/z 133 from a GA patient sample (blue). Panel IV presents the IR spectra of the re-isolated CID fragment (m/z 113, neutral loss of mass 18) from deprotonated glutaric acid (black) and m/z 113, neutral loss of mass 18 from m/z 131 from a GA patient sample (blue)

Having demonstrated the distinction on the basis of IR fingerprints of the two isobaric diacids above from reference standards, we are able to identify unknowns from a sample using the generated reference IR spectra. GA is an IEM for which glutaric acid is a known biomarker and is found in highly elevated levels in patients. In all four panels of Fig. 3, reference IR spectra of the four glutaric acid derived ions are overlaid with IR spectra of ions having corresponding m/z values after ESI of a urine sample from a GA patient, identifying glutaric acid as a biomarker on the basis of four IR fingerprints (four different m/z features all derived from glutaric acid).

### Details of molecular structure through calculated IR spectra and gas phase ion chemistry

**Fig. 4:**
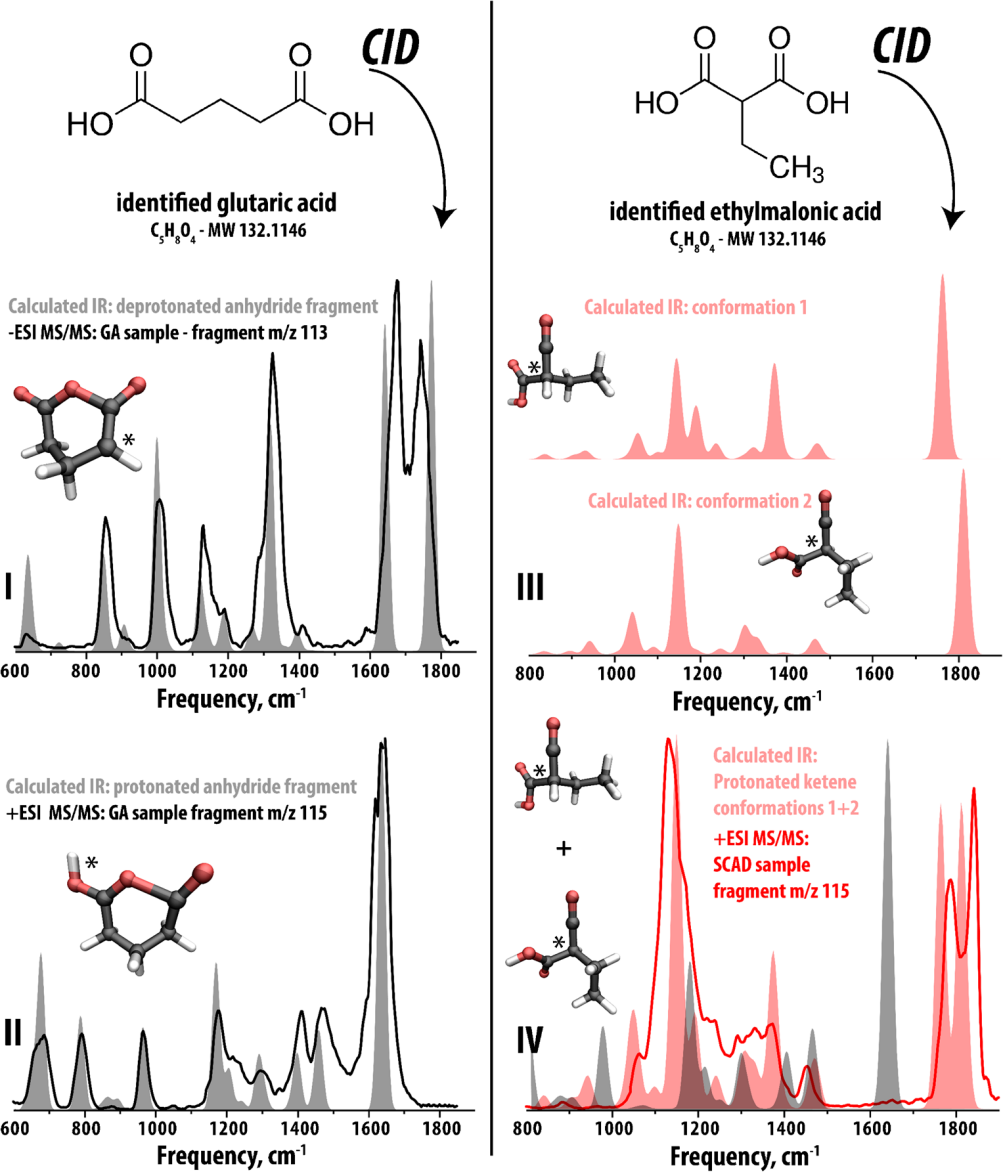
**Identification of molecular structures on the basis of comparison to calculated reference IR spectra.** Panel I presents the IR spectrum of the m/z 113 CID fragment from m/z 131 from a urine sample of a GA patient (-ESI) compared to the calculated IR spectrum (shaded gray) predicted for a candidate structure corresponding to deprotonated glutaric anhydride (structure inlayed). Panel II presents the IR spectrum of the m/z 115 CID fragment from m/z 133 from a urine sample of a GA patient (+ESI) compared to the calculated IR spectrum (gray) predicted for a candidate structure corresponding to protonated glutaric anhydride (structure inlayed). Panel III presents two calculated IR spectra corresponding to two gas phase conformations of a ketene/acid fragment structure for the m/z 115 CID fragment from m/z 133 from a urine sample of a SCAD patient. Panel IV presents the IR spectra from panel II combined and compared to the experimental IR spectrum for the m/z 115 CID fragment from the SCAD sample m/z 133 precursor ion. The calculated spectrum of the isobaric fragment shown in panel II is also compared (gray shaded) showing clear mismatch.”*“s in all structures indicate the site of protonation/deprotonation

The comparison of IR spectra for the collision induced dissociation (CID) fragments in panels III and IV of Fig. 3 in both cases allow assignment of matching fragment ion structures and thus provide additional weight to the precursor ion assignment as glutaric acid (protonated and deprotonated, respectively). However, the measured IR spectra of these fragment ions alone do not reveal their molecular structures, only the presence of particular functional groups (for example, bands around 1700 cm^−1^ indicate a carbonyl group in the structure). Using chemical intuition, the fragmentation mechanism (corresponding to loss of mass 18, i.e., loss of H_2_O) can be hypothesized and IR spectra can be quantum chemically calculated for arbitrary candidate fragment structures. These predicted IR spectra, when matched to the measured spectra, can be used to assign molecular structures to the fragment ions. This comparison is presented in Fig. 4, where we assign ring-closed anhydride structures to the fragments originating from glutaric acid and a ketene/carboxylic acid structure from ethylmalonic acid. These structures are as well consistent with the length of the carbon chain connecting the two acid groups in the respective precursors, as a ring-closed structure is hardly possible to form from ethylmalonic acid.

As an example of how molecular structure/conformation information can be extracted by comparison to calculated IR spectra and their corresponding calculated structures, we focus here on the carbonyl stretching frequency range between 1600 and 1850 cm^−1^ for a detailed analysis. Panel I of Fig. 4 presents the calculated and experimental IR spectra of the m/z 113 CID fragment (precursor m/z 131 assigned as deprotonated glutaric acid from a GA patient sample) with the assigned structure inlayed. In this structure, the carbonyl groups are not equivalent due to deprotonation occurring on the carbon atom adjacent to one of them (indicated with “*”). This results in a strengthening of the C=O bond which shifts one stretching vibration to a higher frequency, splitting the two carbonyl stretching vibrations at approximately 1740 vs. 1675 cm^−1^. Panel II is an analogous comparison of the calculated and experimental spectra for the m/z 115 CID fragment (precursor m/z 133 assigned as protonated glutaric acid from GA patient sample) with the assigned structure once again inlayed. In this structure, protonation occurs on one carbonyl group (indicated with a “*”), causing a shift to lower frequency of the protonated C=O stretching vibration (~1630 cm^−1^) due to a weakening of the C=O bond. The carbonyl that is not protonated is correspondingly strengthened and shifted to higher frequency (outside of the range probed in this particular experiment and thus unobserved, ~2120 cm^−1^). Panel III presents two calculated IR spectra proposed for the m/z 115 CID fragment (precursor m/z 133 assigned as protonated ethylmalonic acid from SCAD patient sample) with the corresponding calculated molecular structures inlayed. In this case, two orientations of the carboxyl group are possible, one in which the carbonyl oxygen interacts with the carbon of the ketene group and the other in which the group is rotated by ~180 degrees and the carbonyl is free from intramolecular interaction. The presence of these two conformations in the trapped ion population causes a splitting of the C=O stretching vibration where the free C=O stretch is found shifted to higher frequency (~1840 vs. 1780 cm^−1^). In both conformations the ketene C=O is shifted to much higher frequency (~2200 cm^−1^) and is unobserved in the frequency range scanned in these experiments. Panel IV presents the calculated IR spectra of the two conformations combined in a 50:50 ratio in shaded red, together accounting for the experimental IR spectrum of the m/z 115 CID fragment (from the m/z 133 precursor ion from the SCAD sample) and providing a convincing second level signature of ethylmalonic acid in the SCAD sample. We note that by confirming the different molecular structures of the CID fragments resulting from H_2_O loss from glutaric acid and ethylmalonic acid, additional confirmation of the precursor ion structures is obtained.

There are several criteria by which we assign a calculated IR spectrum to be a “match” to the experimental one. The output of an IR spectrum calculation is a line spectrum (peak positions without any defined broadening). This makes the alignment of peak center positions between experiment and theory the most important criteria instead of the peak area (to facilitate comparison with experiment we impose a broadening of ~25 cm^−1^ using a Gaussian line shape of the calculated line spectra). Calculated intensities correspond only to the process of a single IR photon absorption, whereas we use multiple photon dissociation to detect IR photon absorption. As a result, correlation between calculated IR absorption intensities and observed experimental IR dissociation yields can deviate, though often still match favorably. In panel IV of Fig. 4, the calculated IR spectrum of the protonated anhydride m/z 115 fragment from panel II is included as a gray trace. Though corresponding to an isobaric ion structure with similar functionality, clear distinction is observed in comparison to the IR spectra of the m/z 115 fragment from the SCAD sample (red line).

## Discussion

### Identification of functional groups

**Fig. 5:**
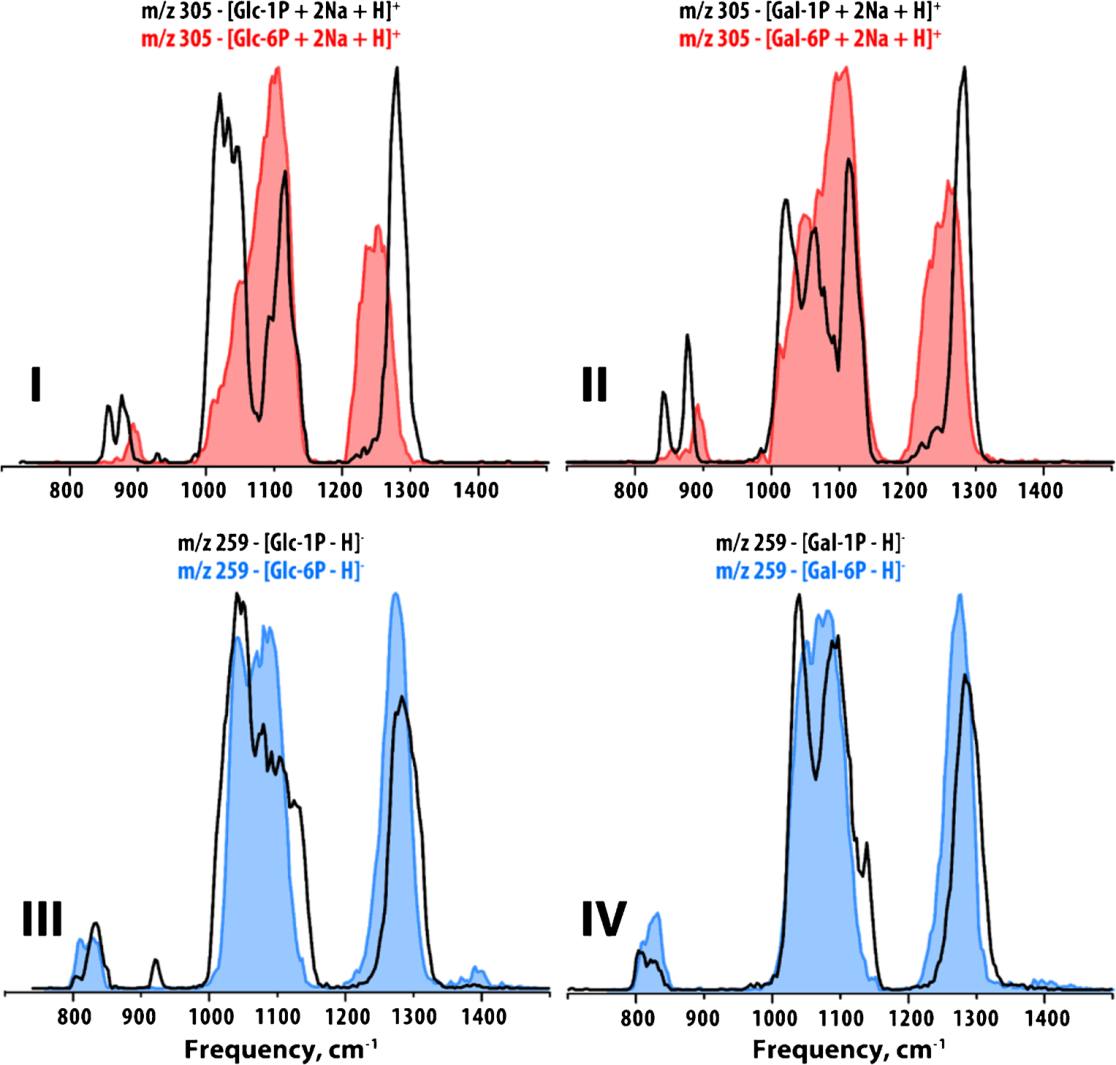
**IR spectra of phosphorylated glucose and galactose.** Panels I and II compare the IR spectra of glucose 1- and 6-phosphate and galactose 1- and 6-phosphate in +ESI as singly charged protonated disodium ions. Panels III and IV compare the IR spectra of glucose 1- and 6-phosphate and galactose 1- and 6-phosphate in –ESI as singly charged deprotonated ions

**Fig. 6:**
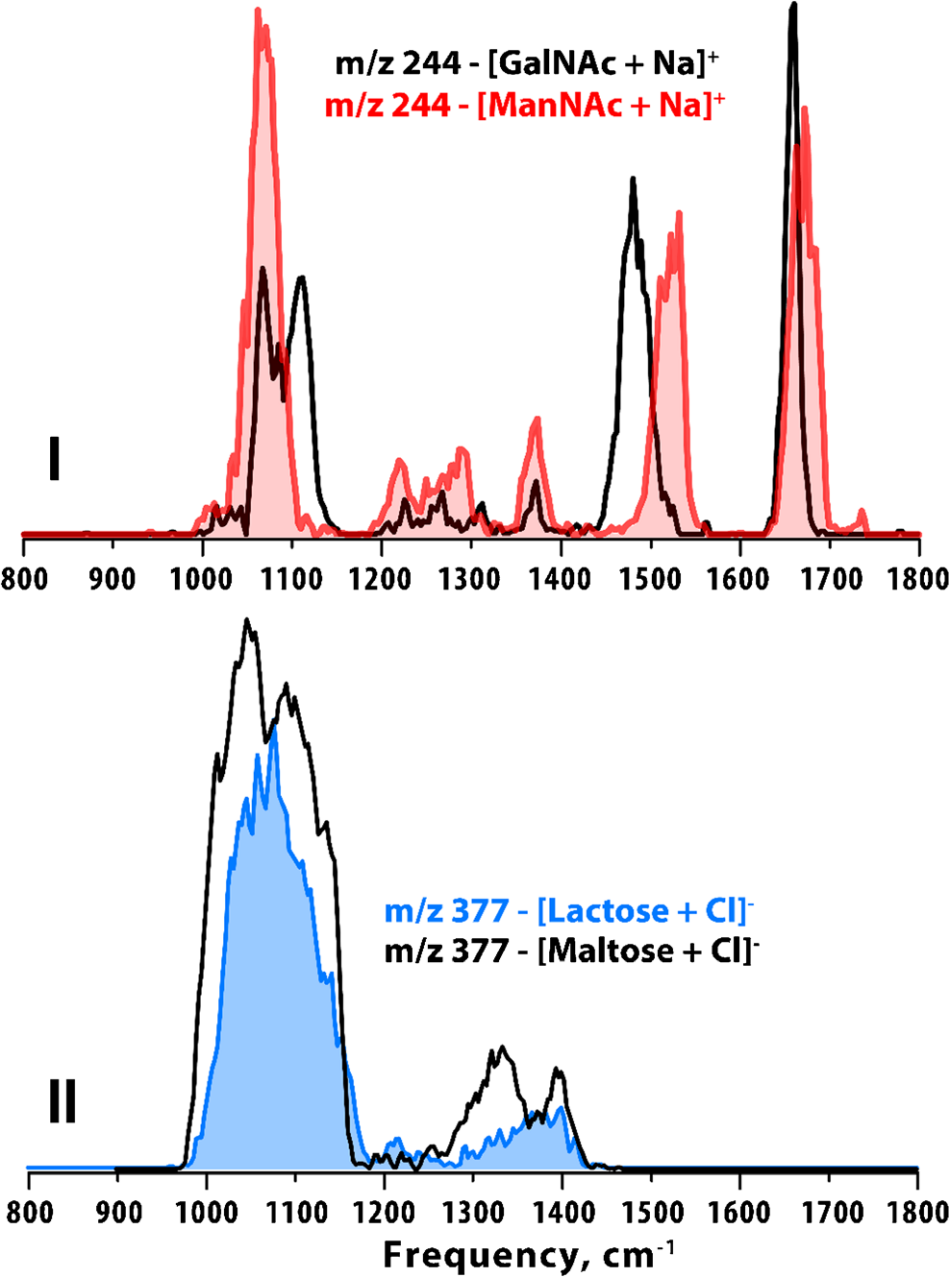
**Comparison of the IR spectra of different saccharides.** Panel I compares the IR spectra of N-acetylated mannose and galactose as sodium adducts in +ESI. Panel II compares the IR spectra of the unfunctionalized disaccharides lactose and maltose as chloride adducts by –ESI

Figure 5 presents a comparison of IR spectra of the phosphorylated hexoses, glucose and galactose, distinguishing phosphorylation in the 1 position or 6 position in both +ESI (top panels) and –ESI (bottom panels). In all IR spectra, the intense feature between ~1200–1300 cm^−1^ is dominated by vibrations of the phosphate functional groups (compared to the IR spectra in Fig. 6 for two sets of saccharides that are not phosphorylated). Clearly, the position of the phosphorylation has an effect on the gas-phase conformations of these ions that results in significant differences in their IR spectra, making them readily distinguishable. Figure 6 compares, in panel I, the IR spectra of N-acetylgalactosamine and N-acetylmannosamine and in panel II the IR spectra of two unfunctionalized disaccharides, lactose and maltose. In addition to distinguishing these challenging sets of isobaric metabolites, we can also attribute specific IR spectral features to particular functional groups within these different classes of saccharides. For the N-acetylhexosamines, two bands above 1400 cm^−1^ (~1500 cm^−1^ is the C-N vibration and ~1650 cm^−1^ is the C=O stretch) are present that are absent for unfunctionalized saccharides and phosphorylated saccharides. In Fig. 5, as noted above, a very intense feature between 1200 and 1300 cm^−1^ (bands of the phosphate group) is observed that is absent in the IR spectra for both classes of saccharides presented in Fig. 6. Information on the functional groups present in a molecule, while not sufficiently specific to identify full molecular structures, provides clues on features of molecular structure that are useful when, for example, a database search returns multiple isobaric candidate structures.

### Accessibility and database potential

At the moment, only a few laboratories worldwide have both the MS and mid-IR laser infrastructure to conduct analytical IR-IS experiments. This is largely related to the fact that a majority of the published IR-IS studies have utilized IR free electron lasers (IR-FEL), which are of limited availability. Until recently, alternative mid-IR laser sources, such as optical parametric oscillators (OPO), have only been available with relatively low output power and limited tuning ranges, somewhat limiting their scope of application, though examples of their application to metabolite identification do exist (Martens et al 2017a, b; Gorlova et al 2017). Nevertheless, several IR-FEL facilities (including FELIX) are currently open to external researchers. In addition, with the quickly advancing development of new mid-IR laser sources (such as quantum cascade lasers), the possibilities for benchtop IR-IS techniques in state-of-the-art analytical laboratories specializing in small molecule identification is continually moving ahead. This is especially true for applications that do not require large laser tuning ranges, such as targeted screening experiments. Recent advances in table-top IR lasers produced commercially available IR-IS suitable lasers capable of tuning over ~10–200 cm^−1^ centered anywhere in the vibrational fingerprint region (600–3800 cm^−1^). To facilitate developments in this directly, our group is pursuing the inclusion of IR-IS generated IR spectra for a series of well-known challenging isobaric metabolites in the human metabolome database (HMDB) (Wishart et al 2013). The first sets of isobaric metabolites we generated IR spectra for to be included in the database are presented in Table 1. These results illustrate the wide range of chemical compounds that are distinguishable by their IR spectra. Including full reference IR spectra (generated using the IR-FEL) over the entire vibrational fingerprint region will certainly guide the advance of IR-IS using table-top infrared lasers with limited tuning range in the future by identifying spectral regions that are selective to one (or a set of) metabolite ion(s). A feature of IR spectroscopy is that vibrational line positions (peak centers) for an arbitrary molecule are closely reproducible irrespective of the experimental apparatus that is used (MS or IR laser). This makes IR spectra valuable chemical properties for inclusion in the HMDB as the reproducibility of IR-IS measurements has the potential to be favorable between labs. Infrared spectroscopy primarily probes functional groups and is highly-sensitive to variations in intramolecular interactions. For an unknown mass peak, some basic chemical information (functional groups) can be directly inferred. In an approach similar to the conventional application of IR spectroscopy in organic chemistry, bands at specific wavelengths can be tied to the presence of certain functional groups. For example, a vibrational peak between 1750 cm^−1^ and 2000 cm^−1^ is indicative of a carbonyl group, as well as that it is not involved in an intramolecular hydrogen bond; the presence of OH and NH groups can be readily identified by the stretching vibrations between 2900 and 3800 cm^−1^. This information can be of significant value when one considers multiple candidate structures from a database search related to an unknown m/z feature. Additionally, by demonstrating examples of metabolites that are challenging to distinguish by standard LC/MS approaches, but distinguishable by IR-IS, researchers worldwide can submit applications for IR-IS experiment time at IR-FEL user laboratories for measurements that will answer their specific research questions.

**Table 1:** Illustrative examples of metabolites for which IR spectra have been measured using the IR-IS technique at the FELIX Laboratory

	MW (neutral)	Compound	Source	Class	ID by IR-IS?
				organic acids	**✓**
1	168.1467	3,4-dihydroxyphenylacetic acid	secondary to medication		
2	168.1467	2,5-dihydroxyphenylacetic acid	alkaptonuria		
				N-acetylhexosamines	**✓** (Martens et al 2017b)
3	221.2078	N-acetylglucosamine	metabolite		
4	221.2078	N-acetylgalactosamine	metabolite		
5	221.2078	N-acetylmannosamine	NANS-deficiency		
				acylglycines	**✓**
6	207.2258	N-acetyl-L-phenylalanine	PKU		
7	207.2258	phenylpropionylglycine	MCAD		
				prostaglandins	**✓**
8	352.4651	prostaglandin E2	metabolite		
9	352.4651	prostaglandin D2	metabolite		
				organic acids	**✓**
10	118.088	methylmalonic acid	MMA		
11	118.088	succinic acid	metabolite, oncometabolite		
				organic acids	**✓**
12	132.1146	ethylmalonic acid	SCAD		
13	132.1146	glutaric acid	GA(T1)		
				phosphosaccharides	**✓**
14	260.1358	glucose 1-phosphate	metabolite		
15	260.1358	glucose 6-phosphate	metabolite		
16	260.1358	galactose 1-phosphate	metabolite		
17	260.1358	galactose 6-phosphate	metabolite		
				disaccharides	**✓**
18	342.2965	lactose	metabolite		
19	342.2965	maltose	metabolite		
				statins	**✓** (Martens et al 2017a)
20	574.6392	ortho-hydroxyatorvastatin	drug metabolite		
21	574.6392	para-hydroxyatorvastatin	drug metabolite		

**Fig. 7:**
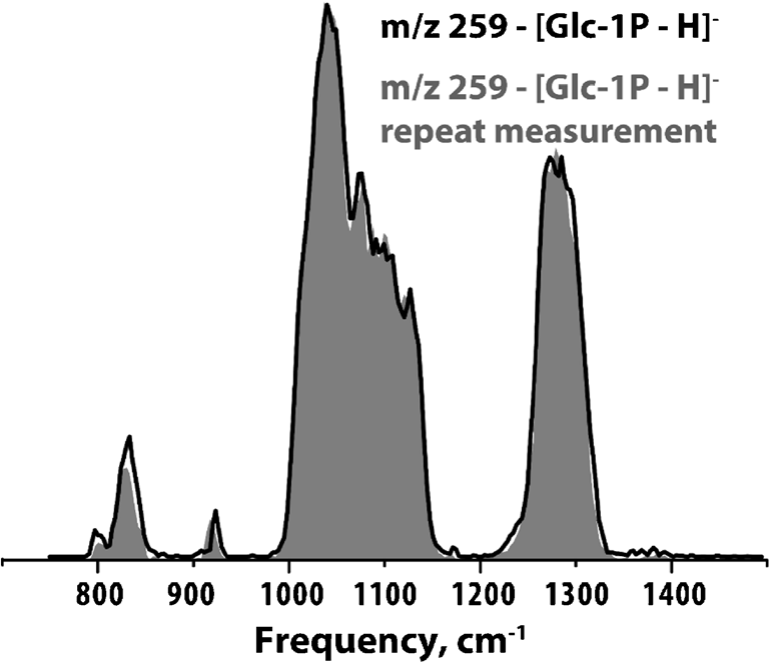
**IR spectra of glucose 1-phosphate.** Two IR spectra of deprotonated glucose 1-phosphate were measured in duplicate under identical experimental conditions, demonstrating reproducibility of IR peak area over the entire spectral range of approximately 97%

Of course, the database potential of an experimental dataset depends highly on reproducibility. Figure 7 illustrates the favorable reproducibility of IR-IS measured peak areas (better than 97%), over two repeated scans (such reproducibility has previously been demonstrated throughout the nM to mM concentration range (Martens et al 2017b)). Note that by considering the fragmentation yield as the IR intensity (see Methods section), we compare the fraction of ions that dissociate at each frequency point, making the measurements independent of the total ion count and hence of concentration. Currently, the measurement of one IR spectrum typically takes ~10 min using ESI flow rates of ∼2 μL/min. At nM concentrations, this corresponds to sample consumption on the order of a few nanograms per IR spectrum.

### Outlook

A number of groups, including our own at the FELIX laboratory, are actively pursuing the combination of mass spectrometry and IR spectroscopy as an analytical tool (Martens et al 2017a, b; Cismesia et al 2016; Gorlova et al 2017). In our group, this work is currently focused on the following three primary objectives: 1) optimizing the efficiency of IR spectra acquisition so that an IR spectrum (perhaps over a limited spectral range of a few hundred wavenumbers) can be acquired in less than several 10s of seconds, 2) optimizing the sensitivity of the mass spectrometry aspect of the IR-IS experiment (currently low nanomolar for setups based on commercial MS platforms) for maximum possible coverage of physiologically relevant concentrations, and 3) combining all aspects of the IR-IS experiment together with HPLC protocols for the routine analysis of complex mixtures such as bio-fluids. Our group has recently demonstrated advances in sensitivity and efficiency that combine IR-IS with offline (fraction collection) HPLC (Martens et al 2017a) and are actively pursuing online HPLC/IR-IS schemes using either a stop flow approach or piecewise spectral acquisition of several hundred wavenumber (cm^−1^) intervals over the desired spectral domain.

## Abbreviations

(U)HPLC/MS^(n)^: (ultra) high performance liquid chromatography/high-resolution (tandem) mass spectrometry
NMR: nuclear magnetic resonance spectroscopy
μM: micromolar
nM: nanomolar
m/z: mass/charge
IR-IS: infrared ion spectroscopy
MS: mass spectrometry/mass spectrometer
IEM: inborn error of metabolism
GA: glutaric aciduria
SCAD: short-chain acyl-CoA dehydrogenase deficiency
ESI: electrospray ionization
CID: collision induced dissociation
IR-FEL: infrared free electron laser
FELIX: free electron laser for infrared experiments
OPO/OPA: optical parametric oscillator/amplifier
HMDB: human metabolome database
FWHM: full width at half maximum
MP2: second order Møller–Plesset perturbation theory
